# Gene Expression Analysis of Toll-Like Receptor Pathways in Heterophils from Genetic Chicken Lines that Differ in Their Susceptibility to *Salmonella enteritidis*

**DOI:** 10.3389/fgene.2012.00121

**Published:** 2012-07-04

**Authors:** Michael H. Kogut, Hsin-I Chiang, Christina L. Swaggerty, Igal Y. Pevzner, Huaijun Zhou

**Affiliations:** ^1^Southern Plains Agricultural Research Center, Agricultural Research Service, United States Department of AgricultureCollege Station, TX, USA; ^2^Department of Poultry Science, Texas A&M UniversityCollege Station, TX, USA; ^3^Cobb-Vantress, Inc.Siloam Springs, AR, USA; ^4^Department of Animal Science, University of CaliforniaDavis, CA, USA

**Keywords:** Toll-like receptors, heterophils, microarray, chickens, genetic selection

## Abstract

Previously conducted studies using two chicken lines (A and B) show that line A birds have increased resistance to a number of bacterial and protozoan challenges and that heterophils isolated from line A birds are functionally more responsive. Furthermore, when stimulated with Toll-like receptor (TLR) agonists, heterophils from line A expressed a totally different cytokine and chemokine mRNA expression pattern than heterophils from line B. A large-scale gene expression profile using an Agilent 44K microarray on heterophils isolated from line A and line B also revealed significantly differential expression in many immune-related genes following *Salmonella enteritidis* (SE) stimulation, which included genes involved in the TLR pathway. Therefore, we hypothesize the differences between the lines result from distinctive TLR pathway signaling cascades that mediate heterophil function and, thus, innate immune responsiveness to SE. Using quantitative RT-PCR on mRNA from heterophils isolated from control and SE-stimulated heterophils of each line, we profiled the expression of all chicken homologous genes identified in a reference TLR pathway. Several differentially expressed genes found were involved in the TLR-induced My88-dependent pathway, showing higher gene expression in line A than line B heterophils following SE stimulation. These genes included the TLR genes *TLR4*, *TLR15*, *TLR21*, *MD-2*, the adaptor proteins *Toll-interleukin 1 receptor domain-containing adaptor protein* (*TIRAP*), *Tumor necrosis factor-receptor associated factor 3* (*TRAF3*), the IκB kinases transforming growth factor-β*-activating kinase 1* (*TAK1*), *IKK*ε *and IKK*α, the transcription factors *NFkB2 and interferon regulatory factor 7*, *phosphatidylinositol-3 kinase* (*PI-3K*), and the mitogen-activated protein kinase *p38*. These results indicate that higher expression of TLR signaling activation of both MyD88-dependent and TRIF-dependent pathways are more beneficial to avian heterophil-mediated innate immunity and a complicated regulation of downstream adaptors is involved in stronger induction of a TLR-mediated innate response in the resistant line A. These findings identify new targets for genetic selection of chickens to increase resistance to bacterial infections.

## Introduction

Host genetics plays an indispensable role in response to *Salmonella* colonization of chickens. For the past several years, we have been profiling the phenotype of two parental broiler lines (A and B) with regard to their resistance or susceptibility against bacterial (*Salmonella enteritidis*, Ferro et al., 2004; Swaggerty et al., 2005a; *Enterococcus gallinarum*, Swaggerty et al., 2005b; *Campylobacter jejuni*, Li et al., 2008; and protozoan *Eimeria*, Swaggerty et al., 2011) challenges. In all cases, line A chickens are more resistant to the pathogen challenges than line B chickens. Mechanistically, this resistance was mediated by the predominant avian granulocyte, the heterophil, with heterophils from Line A functionally more responsive and capable of producing a differential cytokine/chemokine profile compared with line B (Ferro et al., 2004; Swaggerty et al., 2004, 2005a). However, we focused all of these studies on downstream events and/or end products (cell effector functions and cytokine/chemokine gene expression), which led us to ask whether the differences were initiated at either the level of receptor recognition or downstream signaling events induced by ligation of the receptors.

Recognition of potential pathogenic microbes by the innate immune system is the function of a class of cellular receptors known as pattern-recognition receptors (PRRs), which include Toll-like receptors (TLRs). The TLR superfamily represents an evolutionarily conserved signaling system that is a decisive determinant of the innate immune and inflammatory responses. The innate system uses these germ-line encoded receptors to detect evolutionarily conserved microbial proteins, lipids, and nucleic acids (microbial-associated molecular patterns, MAMPs; Fearon and Locksley, 1996). Microbial product-induced activation leads to the activation of intracellular signaling pathways that initiate microbial killing mechanisms, the production of pro- and/or anti-inflammatory cytokines, and up-regulation of co-stimulatory molecules required for antigen presentation to the acquired immune system (Medzhitov and Janeway, 1997). A broad TLR expression profile has been reported in heterophils which suggest that heterophils may play a major role as first-line effector cells through the TLR-induced signaling pathway (Kogut et al., 2005, 2006).

Toll-like receptors are evolutionarily conserved microbial sensing receptors that are able to detect microbial lipids, proteins, and nucleic acids (Takeuchi and Akira, 2010). The avian genome encodes 10 functional TLRs that are located either on the cell surface or within endosomes (Brownlie and Allan, 2011). TLRs located on the cell surface induce the transcriptional activation of pro-inflammatory cytokines, chemokines, and antimicrobial proteins and are mediated by nuclear factor-κB (NF-κB) and mitogen-activated protein kinase (MAPK) pathways through initial activation of the adaptor protein Myeloid differentiation factor 88 (MyD88). Receptor engagement induces MyD88 to activate IL-1R associated kinase-4 (IRAK4), which in turn activates other IRAK family members. These IRAK members then activate the E3 ubiquitin protein ligase TNFR-associated factor 6 (TRAF6) that links with members of an E2 ubiquitin-conjugating enzyme complex. Following a series of ubiquitination steps, transforming growth factor-β (TGF-β)-activated kinase 1 (TAK1) complexes with TAK1-binding proteins that then activates the IκB kinase (IKK) complex and MAPK pathway. The IKK complex phosphorylates and ubiquitinates the NF-κB inhibitor IκBα, marking it for degradation. NF-κB is released from IκBα and translocates to the nucleus to initiate the expression of pro-inflammatory cytokines (O’Neill, 2008; Takeuchi and Akira, 2010). The MAPK pathway activates the transcription factor activator protein-1 (AP-1), which is also responsible for the expression of the pro-inflammatory cytokines.

With all of this information at hand, we hypothesized that the differences between the two genetic lines of chickens result from distinctive TLR recognition and/or signaling pathway cascades that mediate heterophil function and; thus, innate responsiveness to various bacterial and protozoan infections in chickens. Therefore, using a chicken genome Agilent microarray and quantitative real-time PCR (qRT-PCR) analysis, we evaluated TLR pathway gene expression differences between heterophils from the two lines of chickens with and without infection with *S. enterica* serotype Enteritidis (SE).

## Materials and Methods

### Experimental chickens

The two distinct parental broiler lines used in this study were obtained from a commercial company. To maintain confidentiality, the lines were designated as A and B. At the day of hatch, chickens were placed in floor pens (8 feet × 8 feet) containing wood shavings, provided supplemental heat, water, and a balanced, unmedicated corn and soybean meal based chick starter diet *ad libitum*. The feed was calculated to contain 23% protein and 3200 kcal metabolized energy/kg of diet, and all other nutrient rations met or exceeded the standards established by the National Research Council (1994).

### Bacteria

A poultry isolate of SE (#97-11771) was obtained from the National Veterinary Services Laboratory (Ames, IA, USA). SE was cultured in tryptic soy broth (Difco Laboratories, Becton Dickinson, Co., Sparks, MD, USA) overnight at 41°C. Stock SE (1 × 10^9^ cfu/ml) was prepared as previously described (Kogut et al., 2010).

### Heterophil isolation

Heterophils were isolated from the peripheral blood of 100 chickens per line 6 days post-hatch. Following blood collection, heterophils were isolated as previously described (Kogut et al., 2012). Briefly, blood from chickens was collected in vacutainer tubes containing disodium ethylenediaminetetraacetic acid (EDTA; BD vacutainer, Franklin Lakes, NJ, USA) and mixed thoroughly. The blood and EDTA for each line was pooled and diluted 1:1 with RPMI 1640 media containing 1% methylcellulose and centrifuged at 40 × *g* for 15 min at 4°C. The supernatant was transferred to a new conical tube and diluted with Ca^2+^- and Mg^2+^-free Hanks balanced salt solution (1:1), layered onto discontinuous Histopaque^®^ gradients (specific gravity 1.077 over 1.119) and centrifuged at 190 × *g* for 1 h at 4°C. The Histopaque^®^ layers were collected, washed with RPMI 1640 (1:1) and pelleted at 485 *g* for 15 min at 4°C. The cells were then re-suspended in fresh RPMI 1640, counted on a hemacytometer, and diluted to 1 × 10^7^/ml in RPMI. All tissue culture reagents and chemicals obtained from Sigma Chemical Company, St. Louis, MO, USA, unless noted otherwise.

### Total RNA isolation

Heterophils (1 × 10^7^) were treated with 300 μl RPMI or SE (1 × 10^9^ cfu/ml), for 1 h at 39°C on a rotary shaker. Treated heterophils were pelleted, washed with RPMI (485 × *g* for 15 min at 4°C), the supernatant discarded, the cells re-suspended in lysis buffer (Qiagen RNeasy mini RNA extraction kit, Qiagen, Inc., Valencia, CA, USA), and frozen. The lysed cells were transferred to QIAshredder homogenizer columns and centrifuged for 2 min at ≥8000 × *g*. Total RNA was extracted from the homogenized lysate according to the manufacturer’s instructions, eluted with 50 μl RNase-free water and stored at −80°C.

### Microarray experiment design

A dual color, balanced design was used to provide four different comparisons: line A infected (AI)/AC, line B infected (BI)/BC, AC/BC, and AI/BI (C, non-infected controls; I, SE-infected). Only the between line comparisons are reported here; i.e. AC/BC and AI/BI. Within line comparisons have been describe previously (Chiang et al., 2008). Four biological replicates were conducted in each comparison and the dye balance was used throughout in order to prevent the dye bias during the sample labeling.

### Labeling and hybridization

The integrity of total RNA samples was confirmed using Agilent Bioanalyzer 2100 Lab-on-chip system (Agilent Technologies, Palo Alto, CA, USA). Five hundred nanograms (ng) of total RNA were reverse-transcribed to cDNA during which a T7 sequence was introduced into cDNA. T7 RNA polymerase-driven RNA synthesis was used for the preparation and labeling of RNA with Cy3 (or Cy5) dye. The fluorescent cRNA probes were purified using Qiagen RNeasy Mini Kit (Qiagen, Inc., Valencia, CA, USA), and an equal amount (825 ng) of Cy3 and Cy5 labeled cRNA probes were hybridized on a 44 K chicken Agilent array (GEO accession: GSE9416). The hybridized slides were washed using a commercial kit package (Agilent Technologies, Palo Alto, CA, USA) and then scanned using Genepix 4100A scanner (Molecular Devices Corporation, Sunnyvale, CA, USA) with the tolerance of saturation setting of 0.005%.

### Microarray data collection and analysis

For each channel, the median of the signal intensity and local background values were used. A Locally Weighted Linear Regression (LOWESS) normalization was applied to remove signal intensity-dependent dye bias for each array using R program. The normalized data was analyzed using SAS 9.1.3 (SAS Institute, Inc., Cary, NC, USA) with mixed model analysis. The mixed model used to identify significantly differentially expressed genes was:

$${\text{Y}}_{\text{ijklm}}=\mu +{\text{T}}_{\text{i}}\text{ + }{\text{L}}_{\text{j}}\text{ + }{\text{D}}_{\text{k}}\text{ + }{\text{S}}_{\text{1}}{\text{ + T}}^{*}{\text{L}}_{\text{ij}}\text{ + }{\text{e}}_{\text{ijklm}}$$

Where Y_ijklm_ represents each normalized signal intensity; μ is an overall mean value; T_i_ is the main effect of treatment (SE infection) i; L_j_ is the main effect of chicken line j; D_k_ is the main effect of dye k; S_l_ is the random effect of slide l; T*_Lij_ is the interaction between treatment and line; and e_ijklm_ is a stochastic error (assumed to be normally distributed with mean 0 and variance σ^2^). An approximate F test on least-square means was used to estimate the significance of difference for each gene in each comparison where *P* < 0.001 was considered to be statistically different. The false discovery rate (*Q* value) was calculated for each *P*-value using R program according to the method described by Storey and Tibshirani (2003).

### Quantitative real-time PCR

Genes having more than one probe with inconsistent gene regulation expression were further confirmed by qRT-PCR. The anti-coagulated blood from 100 chickens/line was pooled, and the heterophils were isolated from each line as described above. A total of three separate heterophil isolations were made for separate pools of replicated qRT-PCR. The qRT-PCR assay was conducted three times with pooled heterophils (heterophils pooled from 100 chickens from each line with or without SE). At least three replicates were conducted for each gene with the heterophils from each pool of chickens. The data from these three repeated experiments were pooled for presentation and statistical analysis. Total RNA (300 ng) from each sample, AI, line A non-infected (AN), BI, and line B non-infected (BN), were used for cDNA synthesis with random hexamer primer of a Thermoscript RT-PCR system kit (Invitrogen, Carlsbad, CA, USA) according to the manufacturer’s manual. The cDNAs were quantified by qRT-PCR using ABI prism 7900HT system (Applied Biosystems) with SYBR Green PCR Master Mix (Applied Biosystems). The specific oligonucleotide primers (Table 1) were designed by the PRIMER3 program (Rozen and Skaletsky, 2000). The conditions of qRT-PCR amplification were: 1 cycle at 95°C for 10 min, 40 cycles at 95°C for 15 s, and 59°C for 1 min. The chicken β-actin gene was used as the internal control. Dissociation curves were performed at the end of amplification for validating data quality. Each individual sample was run in triplicate and the average critical threshold cycle (Ct) was used for calculating relative quantification by fold change and statistical significance. Data analysis was conducted by two tailed, paired Student’s *t*-test using Microsoft^®^ Excel 2003 version (Microsoft Corporation, 2003). The *P* < 0.05 was considered significant.

**Table 1:** **Primers used for qRT-PCR**.

Gene name	Accession no.	Primer sequence (5′–3′)
β-Actin	NM_205518	F^a^: ACGTCTCACTGGATTTCGAGCAGG
		R^b^: TGCATCCTGTCAGCAATGCCAG
TLR1-1	AY633574	F: CTGTCTTGCCAATCTGTC
		R: GTGAAGGCTCCGTGTATT
TLR1-2	NM_001098854	F: AGCTGCAGGACTTCCTGCGC
		R: TTGTCTGCGTCCACTGCCAC
TLR2-1	AB050005	F: TTAAAAGGGTGTCCAGGAG
		R: GTCCAAACCCATGAAAGAGC
TLR2-2	AB046533	F: AGGCACTTGAGATGGAGCAC
		R: CCTGTTATGGGCCAGGTTTA
TLR3	CR407213	F: CTGCTGCTTCCTTCGTAAGT
		R: GCCAAACAGATTTCCAATCG
TLR4	NM_001030693	F: TGCACAGGACAGAACATCTCTGGA
		R: AGCTCCTGCAGGGTATTCAAGTGT
TLR5	CR353090	F: CTCACCTCTCTCTCAGGGTTTT
		R: TGGGTACACACAGTACCTGTCA
TLR7	AJ720504	F: CCTCGATCTCAACCCTACTTCT
		R: CAGTATCTTTTCCTCACCACACA
TLR15	NM_001037835	F:GTTCTCTCTCCCAGTTTTGTAAATAGC
		R: GTGGTTCATTGGTTGTTTTTAGGAC
TLR21	NM_001030558	F: AGAAGGTGTCGGAGGATGGTG
		R:GGGCTCCAAATGCTGACTGC
MD2	BX932484	F: TCCATCTGGCACGCTGCTGT
		R: GTCGTCGGTCCCGCTGCAAA
MyD88	NM_001030962	F: AAGTTGGGCCACGACTACCT
		R: CAGAAAGGGTTGTTAAGCACTG
TRIF	EF025853	F: TCAGCCATTCTCCGTCCTCTTC
		R: GGTCAGCAGAAGGATAAGGAAAGC
TIRAP	DQ019929	F: CTCATAGCACCACCAGCCACTC
		R: GGGTAATCCTTCCTGTCAATGTCC
IRAK4	AJ720408	F: AATTGCTTGGTTTCTCAAGTG
		R: GCAATTTCACACCTTGTGTTC
TRAF6	CK607050	F: AGTAAATACGAGTGCCCGATCT
		R: TTAGCGAAGTTGTCTGGAAAAA
TRAF3	BX935958	F: CCAGCTCTCAGCAGCAGGAGACA
		R: TCAGCACGAGGACACGGAAGC
IKKα	AJ720520	F: CTTTCATCTATGGCAACTCCTG
		R: ATGTCCAAACCAAGACGTGAT
IKKε	BU133261	F: GTGGACGTGGTGGCCGACTG
		R: GGCGGTTGTGTCCCCTCTGC
TAK1	CR524033	F: GGGCAAAGCAACTCGCCACT
		R: TGATGTGCCTGGCCGTATTTTTCA
NF-κB2	D16367	F: GGTCGACGATGGCTGTGCGG
		R: GAGGGTCGGTGTGCGTCACC
IRF7	U20338	F: AGACCAACTTCCGCTGCGCC
		R: GGCATCCCCTGTGTGTGCCC
p38	CR339030	F: TTGGTTCCACAACTCCAGCACAG
		R: CCGCATCCAGCACCAGCATGT

*^a^Forward primer*.

*^b^Reverse primer*.

## Results

### Global transcription of TLR gene expression between Lines A and B

All TLR were expressed in non-infected heterophils from both lines of birds, but only TLR4 (Line A, 1.54-fold change) and TLR7 (line B, −0.64-fold change) were differentially expressed (*p* < 0.05; Table 2). Upon infection with SE, there were no significant differences in TLR expression in heterophils between lines A and B with one exception, TLR15 (Table 2) where *TLR* 15 was differentially expressed in the heterophils from line A following infection with SE when compared to heterophils from line B chickens.

**Table 2:** **Fold change of Toll-like receptor genes between heterophils isolated from line A and B chickens using microarray analysis**.

Gene name	Accession no.	AC/BC Fold change	AI/BI Fold change	Description
TLR1 type 1	AJ20806	NS	NS	Toll-like receptor 1 type 1
TLR1 type 2	BU405042	NS	NS	Toll-like receptor 1 type 2
TLR2 type 1	AB050005	NS	NS	Toll-like receptor 2 type 1
TLR2 type 2	AB046533	NS	NS	Toll-like receptor 2 type 2
TLR3	CR407213	NS	NS	Toll-like receptor 3
TLR4	NM_001030693	1.54 ± 0.04	NS	Toll-like receptor 4
TLR5	CR353090	NS	NS	Toll-like receptor 5
TLR7	AJ720504	−0.64 ± 0.01	NS	Toll-like receptor 7
TLR15	BU265392	NS	1.62 ± 0.06	Toll-like receptor 15
TLR21	DQ198090	NS	NS	Toll-like receptor 21

*Positive values mean genes have a higher expression in line A*.

*Negative values mean genes have a higher expression in line B*.

*NS, no significance in gene expression between line A and line B (*p* > 0.05)*.

### Real-time PCR analysis of TLR gene expression between lines A and B

The expressions of TLR on heterophils from each line were verified by qRT-PCR analysis (Table 3). When comparing the non-infected control heterophils between lines, only Line A heterophils showed differentially expressed TLRs, specifically TLR4 and, the chicken specific, TLR21. Upon infection with SE, both TLR4 and TLR21, as well as TLR15 were significantly up-regulated when compared to heterophils from line B chickens.

**Table 3:** **Fold change of Toll-like receptor genes between heterophils isolated from line A and B chickens using qRT-PCR analysis**.

Gene name	Accession no.	AC/BC Fold change	AI/BI Fold change
TLR1 type 1	AJ20806	NS	NS
TLR1 type 2	BU405042	NS	NS
TLR2 type 1	AB050005	NS	NS
TLR2 type 2	AB046533	NS	NS
TLR3	CR407213	NS	NS
TLR4	NM_001030693	2.09 ± 0.08	1.28 ± 0.03
TLR5	CR353090	NS	NS
TLR7	AJ720504	NS	NS
TLR15	BU265392	NS	1.98
TLR21	DQ198090	1.72 ± 0.11	1.54 ± 0.07

*Positive values mean genes have a higher expression in line A*.

*Negative values mean genes have a higher expression in line B*.

*NS, no significance in gene expression between line A and line B (*p* > 0.05)*.

### Global transcription of TLR signaling pathway genes between lines A and B

Using Agilent 44K microarray analysis, we determined the global transcriptome of the TLR signaling pathways between lines A and B before and after infection with SE (Table 4). After analyzing 16 conserved TLR pathway genes identified from the chicken genome, we found only one gene in each line that was differentially expressed in the non-infected heterophils. Specifically, we found that *RIP1* was constitutively up-regulated in non-infected line B heterophils; whereas interferon regulatory factor 7 (IRF7) was the only gene up-regulated in non-infected heterophils from line A chickens. However, upon infection with SE, we found a significant up-regulation in six other TLR signaling pathway genes in the heterophils from line A chickens: *MD-2*, *TIRAP*, *IKK*ε, *NF-*κ*B2*, *p38 MAPK 11*, and *p38 MAPK 12* in addition to *IRF7* when compared to line B heterophils (*p* < 0.01). Only the *RIP1* was found to be significantly up-regulated in the line B heterophils when compared to line A heterophils (*p* < 0.05).

**Table 4:** **Fold change of Toll-like receptor pathway genes between heterophils isolated from line A and B chickens using microarray analysis**.

Gene name *gene description*	Accession no.	AC/BC Fold change	AI/BI Fold change
MD-2	BX932484	NS	1.54 ± 0.04
MyD88	AJ851640	NS	NS
TIRAP *Toll/interleukin 1 receptor (TIR) domain-containing adaptor protein*	BX933959	NS	1.31 ± 0.07
IRAK4	AJ720408	NS	NS
TRAF3 T*umor necrosis factor-receptor associated factor 3*	BX935958	NS	NS
TAK1 *TGF-*β*-activating kinase 1*	CR524033	NS	NS
RIP1 *Receptor-interacting protein-1 serine/threonine kinase*	AB108485	−0.68 ± 0.03	−0.72 ± 0.04
IKKε	BU133261	NS	2.52 ± 0.12
IKKα	M74544	NS	NS
NF-κB1	BU479586	NS	NS
NF-κB2	D16367	NS	1.30 ± 0.10
PI-3K	AJ720776	NS	NS
p38 (MAPK 12)	CR339030	NS	1.71 ± 0.12
IRF7 *interferon regulatory factor 7*	U20338	2.40 ± 0.11	2.03 ± 0.08

*Positive values mean genes have a higher expression in line A*.

*Negative values mean genes have a higher expression in line B*.

*NS, no significance in gene expression between line A and line B (*p* > 0.05)*.

### Real-time PCR analysis of TLR signaling pathway genes between lines A and B

Regulation of the TLR signaling pathway genes were verified by qRT-PCR analysis (Table 5). As observed in the microarray analysis, only the *RIP1* gene was constitutively up-regulated in line B heterophils. Similarly, as seen in the microarray analysis, *IRF7* was constitutively up-regulated in heterophils from line A. However, the qRT-PCR data showed that NF-κB2 was constitutively up-regulated in line A heterophils. As observed in the microarray, upon infection with SE, only the *RIP1* was found to be significantly up-regulated in the line B heterophils when compared to line A heterophils. Likewise, *MD-2*, *TIRAP*, *IKK*ε, *NF-*κ*B2*, *p38 MAPK 11*, *p38 MAPK 12*, *IRF7* were up-regulated in heterophils from line A when compared to line B heterophils. More interestingly, using qRT-PCR analysis, we found five more TLR pathway genes that were up-regulated in line A heterophils (*IRAK4*, *TRAF3*, *TAK1*, *IKK*α, and *PI-3K*) when compared to line B heterophils.

**Table 5:** **Fold change of Toll-like receptor pathway genes between heterophils isolated from line A and B chickens using qRT-PCR analysis**.

Gene name	Accession no.	AC/BC Fold change	AI/BI Fold change
MD-2	BX932484	NS	4.41 ± 0.15
MyD88	AJ851640	NS	NS
TIRAP	BX933959	NS	5.37 ± 0.24
IRAK4	AJ720408	NS	4.88 ± 0.21
TRAF3	BX935958	NS	3.68 ± 0.30
TAK1	CR524033	NS	2.65 ± 0.22
RIP1	AB108485	−1.94 ± 0.44	−2.77 ± 0.27
IKKε	BU133261	NS	3.47 ± 0.19
IKKα	M74544	NS	7.26 ± 0.31
NF-κB1	BU479586	NS	NS
NF-κB2	D16367	1.26 ± 0.08	5.91 ± 0.41
PI-3K	AJ720776	NS	4.43 ± 0.28
p38 (MAPK 12)	CR339030	NS	7.62 ± 0.48
IRF7	U20338	3.79 ± 0.22	7.51 ± 0.35

*Positive values mean genes have a higher expression in line A*.

*Negative values mean genes have a higher expression in line B*.

*NS, no significance in gene expression between line A and line B (*p* > 0.05)*.

## Discussion

There are 10 known avian TLRs (TLR1b, 1b, 2a, 2b, 3, 4, 5, 7, 15, and 21; reviewed in (Brownlie and Allan, 2011). We have shown previously that all 10 TLRs are found on chicken heterophils and can be functionally activated *in vitro* with either TLR agonists or intact bacterial cells (Farnell et al., 2003a,b; He and Kogut, 2003; He et al., 2003; Kogut et al., 2005, 2006, 2008; Nerren et al., 2009, 2010). The results from the present microarray and qRT-PCR analysis confirm that the mRNA for all of the known avian TLRs were constitutively expressed in both genetic lines of chickens with only TLR4 (line A) and TLR7 (line B) differentially up-regulated in the non-infected heterophils between the two lines. However, upon infection with SE, three TLR mRNA were significantly up-regulated in heterophils from line A: TLR4, TLR15, and TLR21. These results are noteworthy for two reasons: (a) this is the first report of an up-regulation of TLR21 in chicken heterophils exposed to *Salmonella* and (b) these results are the first to illustrate a striking difference in *Salmonella* recognition by TLRs in heterophils between these genetically distinct parental broiler lines of chickens. These results establish a genetic paradigm for a coordinated TLR response mechanism of the avian heterophil against *Salmonella*. *Salmonella* has at least four TLR ligands: lipopolysaccharide (LPS), lipoproteins, flagellin, and CpG DNA, which activate TLR4, TLR2, TLR5, and TLR21, respectively. Recognition of *Salmonella* lipoproteins and flagellin is apparently similar between the two lines of chickens since we found no significant differences in mRNA expression for TLR2 and TLR5 between line A and B. The agonist(s) for TLR15 are still unknown at this time, but in an earlier series of experiments, we have shown that intact heat-killed Gram-negative or Gram-positive bacteria, but not known TLR agonists induced a significant increase in TLR15 mRNA expression in heterophils (Nerren et al., 2010). Clearly, recognition of the ligand(s) by TLR15 is critical for inducing downstream signaling against *Salmonella* infection in line A chickens. Shaughnessy et al. (2009) found a transient increase in TLR21 mRNA expression in the cecum of *Salmonella*-infected chickens 6 h, but not 20–48 h after infection. Interestingly, just the opposite was found in TLR15 mRNA expression where a transient decrease was found early (6 h) but a significant increase was measured by 48 h post-infection with *Salmonella*.

We also found an up-regulation in expression of the myeloid differentiation (MD)-2 gene that codes for a protein essential for regulating LPS signaling through TLR4 (Dobrovolskaia and Vogel, 2002). MD-2 binds on TLR4 and then the TLR4-MD-2 complex moves to the cell surface. LPS binds MD-2 triggering changes in MD-2 conformation that are detected by TLR4. Engagement of TLR4 activates intracellular signaling *via* the adapter MyD88 (O’Neill, 2006). These results provide further proof of the role of TLR4 in avian heterophil recognition of SE.

Intact bacteria are capable of activating multiple TLR since they typically express a variety of MAMPs on a given cell. Thus, as observed from the present results, multiple TLRs are engaged by the heterophils from both lines of chickens. However, the integrated balance of TLR4, TLR15, and TLR21 to recognize and activate intracellular signaling events in heterophils from line A presumably dictate the resistant phenotype that we have previously observed (Ferro et al., 2004; Swaggerty et al., 2005a). The results from these experiments support complementary roles by recognizing different MAMPs on SE that induce redundant, but synergistic effector mechanisms previously noted for heterophils from line A chickens (Swaggerty et al., 2004, 2005b; Kogut et al., 2006). These redundant mechanisms for microbial recognition and activation of these heterophil-mediated responses serve to provide the resistant phenotype of line A against diverse pathogen challenges (Ferro et al., 2004; Swaggerty et al., 2005a,b, 2011; Li et al., 2008).

Activated TLRs signaling initiates with the recruitment of TIR-domain-containing adaptor molecules (MyD88, TRIF, TIRAP, IRAK) which act as important messengers to activate downstream kinases (IKK complex, MAPKs, TBK1) and transcription factors (NF-κB, AP-1, IRF3, IFR7), which produce effecter molecules including cytokines, chemokines, inflammatory enzymes such as iNOS and oxidase, and type I interferons (Kawai and Akira, 2010). Overall, there are two types of TLR signaling pathways: MyD88-dependent and TRIF-dependent (Beutler, 2009; Kawai and Akira, 2010). MyD88 signaling has generally been linked to NF-κB and MAPK signaling, whereas TRIF-dependent pathway (MyD88-independent pathway) not only mediates pro-inflammatory cytokine production, but also mediates type I interferon production. Studies have also shown crosstalk and overlap between these two pathways depending on the cell type involved (Beutler, 2009). In mammals, all TLRs except TLR3 utilize MyD88-dependent signaling, whereas TLR4 and TLR3 utilize TRIF-dependent signaling. TLR4 is unique in that it utilizes both MyD88- and TRIF-dependent pathways. The MyD88-dependent pathway requires both MyD88 and TIRAP to activate NF-κB, whereas, TRIF-dependent signaling are controlled by TRIF and TRAM. Whether the absence of TRAM on the chicken genome (Brownlie and Allan, 2011) has an effect on the control of the TRIF-dependent pathway is unknown, but results from the present experiments suggest that both the MyD88-dependent and TRIF-dependent pathways are activated in the heterophils from line A during the interaction with SE. Consequently, the absence of TRAM from the chicken genome does not appear to be detrimental.

**Figure 1:**
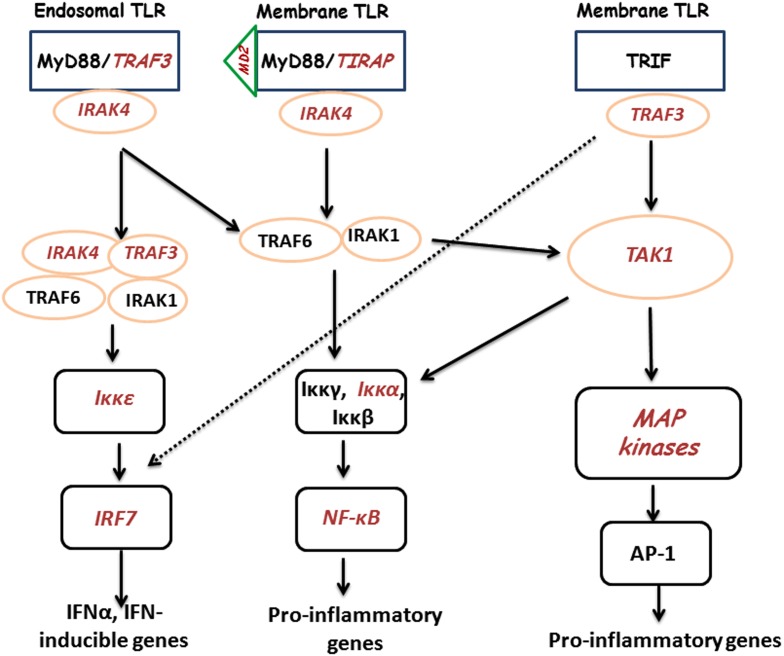
**Cartoon of TLR signaling pathways in heterophils from line A chickens following interaction with SE**. TLRs send signal through TIRAP and MyD88 or TRIF to IRAK/TRAF6 to activate downstream kinases. The signals are delivered to kinases including MAPKs, IKKs, and TBK1, to activate transcription factors, NF-κB, AP-1, and IRF. Transcription factors bind to specific DNA sequences and produce effecter molecules such as cytokines, inflammatory enzymes, chemokines, and type I interferons (IFNs). Components in red italics were significantly up-regulated.

Previously, we demonstrated a large-scale gene expression profiling on heterophils isolated from broilers with different genetic backgrounds (*Salmonella*-resistant line A and -susceptible line B). Many immune-related genes showed significantly differential expression following SE stimulation, which includes genes involved in the TLR pathway (Chiang et al., 2008). In addition, global analysis data suggested a similar TLR regulatory network might exist in both lines where a possible MyD88-independent pathway may participate in the regulation of host innate immunity (Chiang et al., 2008). Therefore, for the present studies, using the mammalian TLR pathway as a reference, an inferred chicken TLR pathway consisting of 72 chicken genes was constructed to compare gene expression between SE-infected to non-infected heterophils from each line. Of these 72 TLR reference genes, we found virtually no significantly differentially expressed genes between lines in the non-infected heterophils. However, upon infection with SE, we found 11 of the TLR reference genes that were significantly up-regulated in heterophils from line A when compared to line B (*MD-2*, *TIRAP*, *IRAK4*, *TRAF3*, *TAK1*, *IKK*ε, *IKK*α, *NF-*κ*B2*, *PI-3K*, *p38*, and *IRF7*). It is evident from the data in these studies that heterophil response from line A birds to SE involves the coordination of genes from *all of the components* of the intracellular TLR signaling pathway: receptors (*TLR4*, *TLR15*, *TLR21*), adaptors (*TRAF3*, *TIRAP*, *IRAK4*), kinases (*I*κκα, *I*κκε, *TAK1*, *p38*), transcription factors (*NF-*κ*B2*, *IRF7*), and effector molecules (*IL-6*, *IL-12A*, *CCL4*, *CCL5*, *IFN-*α). Furthermore, it is evident that SE infection of the line A heterophils induce the activation of both a MyD88-dependent and a TRIF-dependent TLR signaling pathways (Figure 1).

Tumor necrosis factor-receptor associated factor 3 is an important adaptor that transmits upstream activation signals to protein kinases that phosphorylate transcription factors to induce the production of type I IFNs. TRAF3 plays roles in both TLR-dependent and TLR-independent signaling pathways involved in type I IFN production. TIRAP plays a crucial role in MyD88-dependent signal transduction by TLR2 and TLR4, acting as a bridging adaptor to recruit MyD88 (Jenkins and Mansell, 2010). TRAF3 is recruited to TLR adapters, MyD88 and TRIF, and associates with IRF3/7 kinases, TBK1 and IKK-ε, and IRAK1 when these proteins are over expressed in HEK-293T transformed epithelial cells (Oganesyan et al., 2006). Thus, during TLR signaling, TRAF3 serves as a cytoplasmic adapter and transmit upstream signals to downstream kinases involved in type I IFN production. IRAK4 has an essential role in TLR-mediated signaling by associating with MyD88 and induces IRAK1 phosphorylation, recruitment of TRAF6, and engagement of TAK1 (Li, 2008; O’Neill, 2008), which activates MAPKs and transcription factors NF-κB and AP-1, resulting in transcription of genes encoding inflammatory mediators. The kinase activity of IRAK4 plays a critical role in TLR-mediated immune responses. Inactivation of IRAK4 kinase activity leads to (a) reduced mRNA stability and diminished production of cytokines and chemokines in response to LPS stimulation and (b) both TLR7- and TLR9-mediated cytokine and type I IFN production was abolished IRAK4 kinase-inactive knock-in mice (Kim et al., 2007). TAK1 is activated by phosphorylation via TLR2 and/or four-mediated pathway, whose kinase activity is required for NF-κB activation. TAK1 regulates NF-κB-inducing kinase activity that activates IKKα/β downstream of MyD88 and TRAF6. TAK1 is also a MAP kinase kinase kinase for p38 that is critical for the production of pro-inflammatory cytokines (Irie et al., 2000).

Stimulation of TLRs results in the downstream activation of the cytoplasmic Toll/IL-1 receptor (TIR) domain portion of the TLR, which then recruits MyD88/IRAK/TRAF6 and activates the MAPK superfamily cascade (Dalpke and Heeg, 2002; O’Neill, 2002; Akira, 2003) and the transcription factors, NF-κB and AP-1 that leads to the expression of genes that participate in the innate immune response including pro-inflammatory cytokines. The MAPK superfamily of serine/threonine kinases consists of at least three distinct families: p38, extracellular signal-regulated kinase 1/2 (ERK1/2), and c-Jun N-terminal kinase (JNK) that play a major role in cellular activation of a variety of cell types. In mammalian cells, the phosphorylation of the MAPK superfamily has been established as the hallmark of cellular activation following TLR engagement (O’Neill, 2002). We have found that heterophils, when stimulated with specific TLR agonists, activate the p38 and ERK1/2 MAPK signaling cascades leading to the up-regulation of pro-inflammatory cytokine gene expression (Kogut et al., 2005, 2008) and increased responsiveness of line A heterophils were mediated, by an increased ability to the p38 MAPK pathway and specific transcription factors, all of which directly affect the innate immune response (Swaggerty et al., 2011). These results were all confirmed by the present experiments where the gene for *p38* was differentially up-regulated in heterophils from line A heterophils infected with SE when compared to line B heterophils.

RIP1 is an adaptor serine/threonine kinase associated with the signaling complex of death receptors (DRs) including Fas, TNFR1, and TRAIL-Rs which can initiate apoptosis. In addition, RIP1 can bind to TRAF 2 (Hsu et al., 1996) and help recruit IKK (Li et al., 1999; Zhang et al., 2000). RIP1 was found to be significantly down-regulated in line A heterophils when compared to line B heterophils. These results suggest that line A heterophils do not use apoptosis as a immune mechanism to remove *Salmonella* nor do they require RIP1 for NF-κB activation.

IκB kinase and IKK-related kinases play critical roles in regulating the immune response through NF-κB and IFN regulatory factor-dependent signaling transduction cascades. In response to pro-inflammatory stimuli, such as TNFα, IL-1, and TLR agonists (such as LPS), two kinases, TAK1 and mitogen-activated protein/ERK kinase kinase 3, are recruited into the proximity of the IKK complex by interacting with several receptor associated proteins, thereby phosphorylating and activating both IKKα and IKKβ in the cytoplasm. A major consequence of IKKα/IKKβ activation is the initiation of NF-κB-mediated transcriptional activation (Fitzgerald et al., 2003; Hacker and Karin, 2006). Unlike IKKα and IKKβ, which are major catalysts of the NF-κB pathway, IKKε, and TBK1 have restricted functions in the NF-κB activation pathway. They are activated by TLR agonists and viral ssRNA in the cytoplasm and mainly function as mediators of type I IFN gene expression, which contributes to the antiviral response by their activation of the IRF3 and IRF7, which are transcriptional factors with diverse roles in immunity and cellular response to viral infections. Thus, IKKε and TBK1 are important mediators of antiviral response and, together with IKKα and IKKβ, coordinate and organize the host immune defense (Fitzgerald et al., 2003; Hacker and Karin, 2006). IKKα is the other catalytic kinase of the classic IKK complex (along with IKKβ). In contrast to IKKβ’s effect on IκB phosphorylation in the canonical pathway, IKKα might have a crucial function to facilitate NF-κB-dependent gene transcription instead of IκB phosphorylation due to its lower ability to induce IκB phosphorylation. IKKε is the other non-canonical IKK involved in regulating the activation of the IRF3 and NF-κB signaling pathways (Fitzgerald et al., 2003). Upon activation in response to TLR agonists and viral infection, IKKε phosphorylates IRF3 and IRF7 and triggers IRF3/IRF7 nuclear translocation, which results in the up-regulation of type I IFN expressions.

Increasing evidence supports the involvement of the phosphatidylinositol-3 kinase (PI-3K) pathway in the regulation of activation of IRFs by TLRs. The PI-3K pathway can be activated by various TLR ligands and can negatively or positively regulate TLR responses, depending on cell types and the ligands. Both TLR9 and -3 activate the PI-3K/Akt/mTOR pathway leading to the activation of IRF7, -3, and -5 and mTOR kinase activity is required for the interaction between MyD88 and IRF7 (Fukeo and Kayasu, 2003; Cao et al., 2008; Schmitz et al., 2008; Ning et al., 2011). PI-3K is critical for the nuclear translocation of IRF7 and type I IFN production in response to TLR7/9 activation, and mTOR kinase activity is required for the interaction between MyD88 and IRF7 (Schmitz et al., 2008; Ning et al., 2011).

Nuclear factor-κB transcriptional factors are central regulators and transcriptional factors in response to pathogens and viruses. NF-κB transcription factors are important in the regulation of immune and inflammatory responses (Karin and Ben-Neriah, 2000). NF-κB is composed of dimeric complexes of members of the Rel/NF-κB family of polypeptides. This family comprises Rel-A, c-Rel, Rel-B, NF-B1/p50, and NF-B2/p52. NF-κB dimers in non-stimulated cells interact with one of a family of cytoplasmic inhibitory proteins (IκBs) which prevent nuclear entry. This family includes IκB_α_, IBβ and IBε together with the precursor forms of NF-κB1 (p105) and NF-κB2 (p100). NF-κB1 p105 and NF-κB2 p100 are proteolytically processed by the proteasome to produce p50 and p52, respectively. Following agonist stimulation, IκBα, IκBβ, and IκBε and p105 are phosphorylated by the IKK complex, triggering their ubiquitylation and degradation by the proteasome. Associated NF-κB dimers are then released to translocate into the nucleus and modulate gene expression. Proteolysis of NF-κB2 p100 is regulated by the IKKα subunit of the IKK complex, which triggers processing to generate p52 which can then undergo nuclear translocation (Karin and Ben-Neriah, 2000). IRF7 is the master regulator of type I IFNs against pathogenic infections, which activate IRF7 by triggering signaling cascades from PRRs that recognize pathogenic nucleic acids. Activation of IRF7 is a prerequisite for its functions as a transcription factor. Inactive IRF7 resides in the cytoplasm as a latent form when pathogenic infection triggers IRF7 phosphorylation and translocation into the nucleus, where with other co-activators it forms a transcriptional complex that binds to the promoter regions of target genes to activate transcription. Core signaling components involved in signaling cascades leading to activation of IRFs include the adaptors TRIF and MyD88, IRAK1/4, TRAF6, and TRAF3, and the IKKs, IKKε, and TBK1, depending on the activating TLR. IRF7 is activated by pathogenic nuclei acids through pathways mediated by TLR3, -7, and -9 (Ning et al., 2011).

Our results indicate that higher expression of a combination of TLRs and a complicated regulation of downstream adaptors, kinases, and transcription factors are involved in a stronger induction of heterophil-mediated innate immune response; thus, is more beneficial to the resistant line. These findings lay the foundation for future studies on the genetic selection for the regulatory gene network in chicken TLR pathways and immune modulation of SE infection in chickens. Furthermore, the basic TLR signaling pathways regulating innate immunity are central to many infections in poultry. Based on the present and past studies we have conducted to profile the immune gene expression of lines A and B to *Campylobacter* (Li et al., 2010, 2011, 2012), perhaps future studies can be directed toward genotype-specific strategies to control such infections affecting the poultry industry.

## Conflict of Interest Statement

The authors declare that the research was conducted in the absence of any commercial or financial relationships that could be construed as a potential conflict of interest.
